# An Act to Regulate the Practice of Dentistry in Alabama

**Published:** 1887-04

**Authors:** 


*President.*

### An Act to Regulate the Practice of Dentistry in the State of Alabama, as Amended February 28th, 1887.

Section 1. Be it enacted by the General Assembly of Alabama, That from and after the passage of this act, it shall be unlawful for any person to engage in the practice of dentistry in the State of Alabama, unless said person has obtained license from a Board of Dental Examiners, duly authorized and appointed by this act to issue such license; Provided that dentists who have been in the regular practice of dentistry for five years next preceeding the passage of this act shall not be required to submit to an examination, and shall be entitled to license without fee, which shall be transmitted to him by mail, or otherwise, upon his application accompanied by an affidavit to the fact of his having been in the practice for the required time.

Sec. 2. Be it further enacted, That the Board of Dental Examiners shall consist of five (5) dental graduates, or practitioners of dentistry, who have obtained a license to practice dentistry from a Dental Board organized under this act, and who are members in good standing of the Alabama Dental Association: Provided, That said graduates or practitioners have been practicing dentistry in the State of Alabama for a period not less than three (3) years.

Sec. 3. Be it further enacted, That it shall be the duty of said Alabama Dental Association, at its annual meeting in April,

1887, to elect said Board of Dental Examiners, whose terms of office shall be respectively five, four, three, two, and one years, in the order in which they are elected; and at each annual meeting of said Association thereafter, one member shall be elected to fill such vacancy, who shall serve for the period of five years. The President shall have power to fill all vacancies in said Board for unexpired terms.

Sec. 4. Be it further enacted, That it shall be the duty of said Board of Examiners—

1. To meet annually at the time and place of meeting of the Alabama Dental Association, or oftener, at the call of any three members of the Board. Thirty days notice must be given of the time and place of meeting of said Board, said notice to be mailed to all practicing dentists in the State.

2. To prescribe a course of reading for those who study dentistry under private instruction.

3. To grant license to all applicants who undergo a satisfactory examination, who shall pay to the said Board a fee of five dollars for said license.

4. To keep a book in which shall be registered the names of all persons licensed to practice dentistry in this State.

Sec. 5. Be it further enacted, That the book so kept shall be a book of record, and a transcript from it certified to by the officer who has it in keeping, with the common seal of said Board, shall be evidence in any court of this State.

Sec. 6. Be it further enacted, That three members of said Board shall constitute a quorum for the transaction of business, and should a quorum not be present on the day appointed for its meeting, those present may adjourn from day to day until a quorum is present.

Sec. 7. Be it further enacted, That one member of said Board may grant a license for an application to practice until the next regular meeting of the Board, when he shall report the fact, at which time the temporary license shall expire, but such temporary license shall not be granted by a member of the Board after the Board has rejected the applicant.

Sec. 8. Be it further enacted, That any person who shall, in violation of this act, practice dentistry in this State, shall be liable to indictment, and, on conviction, shall be fined not less than fifty nor more than three hundred dollars; Provided, that nothing in this act shall be construed to prevent persons from extracting teeth. Provided that nothing in this act shall be so construed as to require any person who is now lawfully engaged in the practice of dentistry to procure any additional license or to attend any meeting or meetings of the State Dental Association.

Sec. 9. Be it further enacted, That on the trial of such indictment it shall be incumbent upon the defendant, to exempt him from the penalties of this act, to show that he has authority under the law to practice dentistry in this State.

Sec. 10. Be it further enacted, That every person to whom license is issued by said Board of Examiners shall, within thirty days from date thereof, present the same to the Judge of the Probate Court of the County in which he resides, who shall officially endorse said license and seal it with the seal of the Court, and who shall record said license in a book in his office, and who shall be entitled to a fee of one (1) dollar for his services; but a temporary license issued under section 7 of this act need not be sealed or recorded.

Sec. 11. Be it further enacted, That it shall be the duty of the solicitors of this State to prosecute all persons violating all or any portion of this act.

Sec. 12. Be it further enacted, That all laws or parts of laws in conflict with this act be and the same are hereby repealed. [Amended and approved February 28th, 1887.

